# Supplementary material for: Impact of horizontal gene transfer on emergence and stability of cooperative virulence in Salmonella Typhimurium
Source: Nat Commun. 2022 Apr 11;13:1939. doi: 10.1038/s41467-022-29597-7 (PMC9001671; doi:10.1038/s41467-022-29597-7)
Supplement: Supplementary file 1 — Supplementary Information [file 41467_2022_29597_MOESM1_ESM.pdf]

Supplementary information:

Supplementary discussion: Why does inflammation cause only mild *S.*Tm blooms as detected by shedding in Fig. 1?

In our experimental system, we address if HGT can facilitate the emergence of cooperative virulence using *S.*Tm as a model system. *S.*Tm is an excellent candidate to monitor the emergence of cooperative virulence since tractable models for within-host evolution of *S.*Tm (e.g. plasmid transfer and evolution of virulence) have been established that integrate important parameters of the host-pathogen interaction[1-5]. The antibiotic pretreatment model allows robust colonization of *S.*Tm in mice, leading to inflammation that acts as a public good to suppress the microbiota and create a favourable niche for Enterobacteriaceae, such as *S.*Tm, to thrive[3,5]. In the absence of overt inflammation, the microbiota can re-grow and exclude *S.*Tm from the gut[6].

Our experimental approach uses this antibiotic pretreatment model to allow efficient gut luminal colonization of *S.*Tm, a pre-requisite for efficient plasmid exchange. In the experiments in **Fig. 1**, one day after ampicillin pretreatment, two avirulent *S.*Tm strains are introduced into the gut and allowed to grow and exchange plasmids. This experimental setup is unique in the sense that for the first time using this mouse model, we introduce virulence only after plasmid exchange, meaning that virulence can emerge from rare. Virulent clones are initially very low in abundance and only expand to sufficient density to trigger inflammation (~10^8 CFU/g feces[7]) at day 2 post infection (**Fig. S3D**), mediated by plasmid spread. This means that there is a delay of approximately 1-2 days in triggering inflammation, compared to the conventional antibiotic pretreatment model. Microbiota re-growth then likely competes against actively invading *S.*Tm that have recently become virulent, creating a delicate balance that may ultimately prevent overt inflammation. Indeed, the inflammation measured by LCN2 ELISA ranges from 10^1 - 10^3 ng LCN2 / g feces (median ~10^2 ng / g feces at day 3 post infection; **Fig. 1C**), which is strikingly lower than the inflammation triggered in a scenario where virulent clones are present immediately after introduction in the gut (e.g. **Fig. 2B,E**; **Fig. 3A**; **Fig. 4D**; ~10^3 - 10^4 ng LCN2 / g feces at day 1 post infection). The lack of immediate overt inflammation in **Fig. 1C** can explain the absence of clear pathogen blooms (**Fig. 1D**) typically associated with inflammation[5] (i.e., the microbiota starts to re-grow and cannot be suppressed by mild inflammation). This is in contrast to when shedding is maintained in mice where overt inflammation is detected (e.g. **Fig. 2C,F**; **Fig. 3B; Fig. 4E**).

What does this mean for the emergence of virulence by HGT? The intuition for the prevalence of virulence factors in pathogens such as *S.*Tm is that they provide a selective advantage, since HGT-mediated maintenance alone is likely too unstable to be the sole driver for the evolution of virulence factors (**Fig. 1E; Fig. 2D,G; Fig. 3C; Fig. 4G;**[8,9]). However, it is also unlikely that HGT of a virulence-conferring allele permeates the pathogen population immediately to lead to a discernable fitness advantage in the initial host (e.g. pVir spreading to 100% of the population immediately to evoke inflammation-mediated blooms). Therefore, the selective advantage must be elsewhere. Here, we show that pathogen cells that harbour newly acquired virulence determinants can lead to inflammation after transmission, even if inflammation was not overtly triggered in the initial host (e.g. compare inflammation and shedding in **Fig. 1** to **Fig. 2**. Therefore, the lack of pathogen blooms in the first host can be compensated with the inflammation triggered in subsequent hosts after transmission.

Supplementary references:

1 Wotzka, S. Y., Nguyen, B. D. & Hardt, W. D. Salmonella Typhimurium Diarrhea Reveals Basic Principles of Enteropathogen Infection and Disease-Promoted DNA Exchange. *Cell Host Microbe* **21**, 443-454, doi:10.1016/j.chom.2017.03.009 (2017).

2 Bakkeren, E., Diard, M. & Hardt, W. D. Evolutionary causes and consequences of bacterial antibiotic persistence. *Nat Rev Microbiol*, doi:10.1038/s41579-020-0378-z (2020).

3 Barthel, M. *et al.* Pretreatment of mice with streptomycin provides a Salmonella enterica serovar Typhimurium colitis model that allows analysis of both pathogen and host. *Infect Immun* **71**, 2839-2858 (2003).

4 Diard, M. & Hardt, W. D. Evolution of bacterial virulence. *FEMS Microbiol Rev*, doi:10.1093/femsre/fux023 (2017).

5 Stecher, B. *et al.* Salmonella enterica serovar typhimurium exploits inflammation to compete with the intestinal microbiota. *PLoS Biol* **5**, 2177-2189, doi:10.1371/journal.pbio.0050244 (2007).

6 Endt, K. *et al.* The microbiota mediates pathogen clearance from the gut lumen after non-typhoidal Salmonella diarrhea. *PLoS Pathog* **6**, e1001097, doi:10.1371/journal.ppat.1001097 (2010).

7 Ackermann, M. *et al.* Self-destructive cooperation mediated by phenotypic noise. *Nature* **454**, 987-990, doi:10.1038/nature07067 (2008).

8 Dimitriu, T. *et al.* Genetic information transfer promotes cooperation in bacteria. *Proc Natl Acad Sci U S A* **111**, 11103-11108, doi:10.1073/pnas.1406840111 (2014).

9 Nogueira, T. *et al.* Horizontal gene transfer of the secretome drives the evolution of bacterial cooperation and virulence. *Curr Biol* **19**, 1683-1691, doi:10.1016/j.cub.2009.08.056 (2009).

10 Kroger, C. *et al.* An infection-relevant transcriptomic compendium for Salmonella enterica Serovar Typhimurium. *Cell Host Microbe* **14**, 683-695, doi:10.1016/j.chom.2013.11.010 (2013).

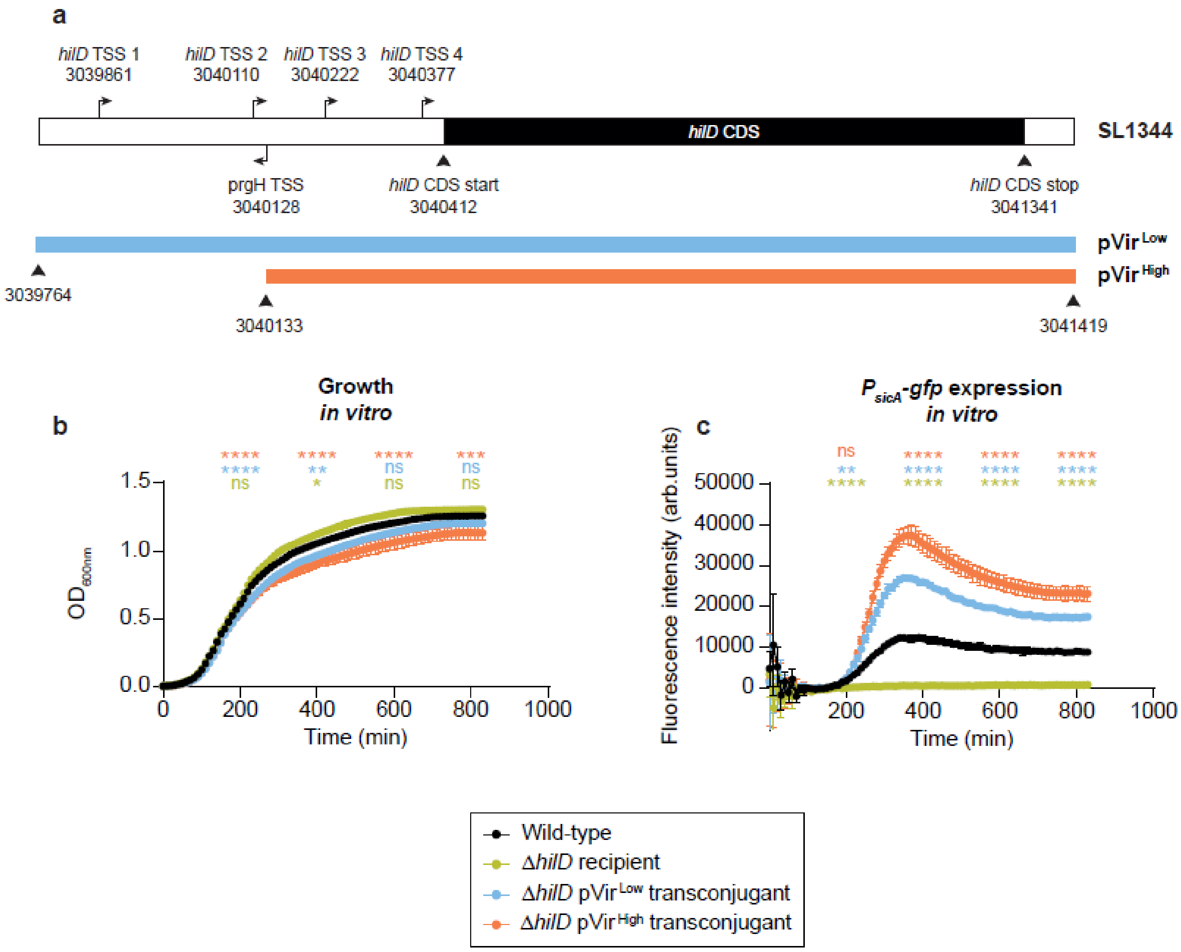

Supplementary Figure S1. Genetic locus of *hilD* cloned into pVir and characterization of *ttss-1* expression and cost *in vitro*. A) The *hilD* coding sequence (CDS) of *S.*Tm SL1344 (NCBI Accession: FQ312003.1), the template for cloning the two pVir constructs, is shown along with the four transcriptional start sites (TSS) of *hilD* identified in[10] (indicated with arrows; the *prgH* TSS is also indicated). The position in the SL1344 genome is indicated numerically. The regions cloned into $pVir^{Low}$ or $pVir^{High}$ are indicated with a green or red bar, respectively. Positions along the 1655 bp genetic locus shown are to scale. **B-C)** *In vitro* analysis of $pVir^{Low}$ (blue) and $pVir^{High}$ (orange) transconjugants compared to a wild-type (black) and a Δ*hilD* mutant (yellow). Strains bearing pM972 (P_{sicA}-*gfp*; TTSS-1 expression reporter) were used to correlate growth (**panel B**) and TTSS-1 expression (**panel C**; arbitrary units). Data is shown as the mean with standard deviation of 2 independent clones for $pVir^{Low}$, 6 independent clones for $pVir^{High}$, 1 clone for wild-type and 1 clone for the Δ*hilD* mutant (3 technical replicates for each clone). A one-way ANOVA with Dunnett's multiple comparisons test is performed at 200, 400, 600, and 800 min post inoculation, comparing each group to the wild-type control (indicated with asterisks corresponding to the colour of the group; p>0.05 (ns), p<0.05 (*), p<0.01 (**), p<0.001 (***), p<0.0001 (****)). Source data are provided as a Source Data file. All strains lacked functional *ttss-2* genes (*ssaV* mutation).

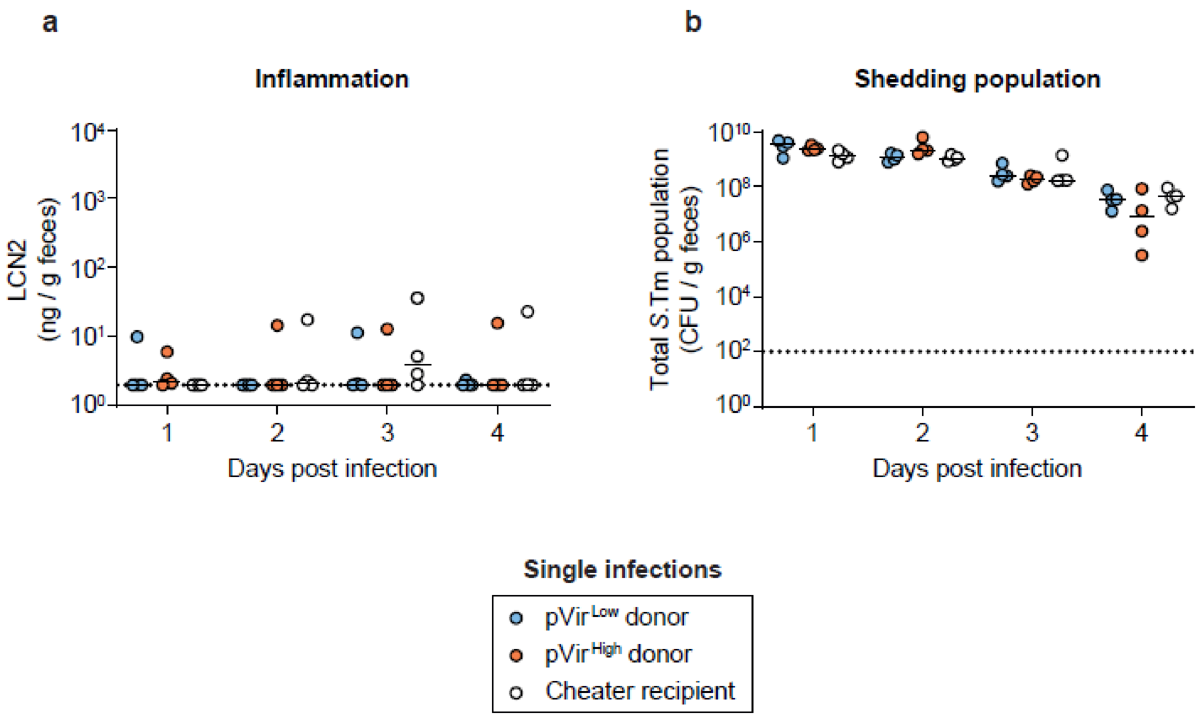

Supplementary Figure S2. The pVir^{Low} and pVir^{High} donors and the recipient cannot trigger inflammation during single-infections. Ampicillin pretreated mice (n=4 per group) were orally infected, either with ~5×10^{7} CFU of pVir^{Low} (blue) donors (14028S Δ*invG* Δ*hilD* Δ*ssaV* pVir), pVir^{High} (orange) donors (14028S Δ*invG* Δ*hilD* Δ*ssaV* pVir), or with recipients (14028S Δ*hilD* Δ*ssaV*; white). All data points are shown and medians are indicated with lines for **A)** Inflammation detected by a LCN2 ELISA and **B)** the total *S.*Tm population quantified by selective plating. Dotted lines indicate the detection limit. Source data are provided as a Source Data file.

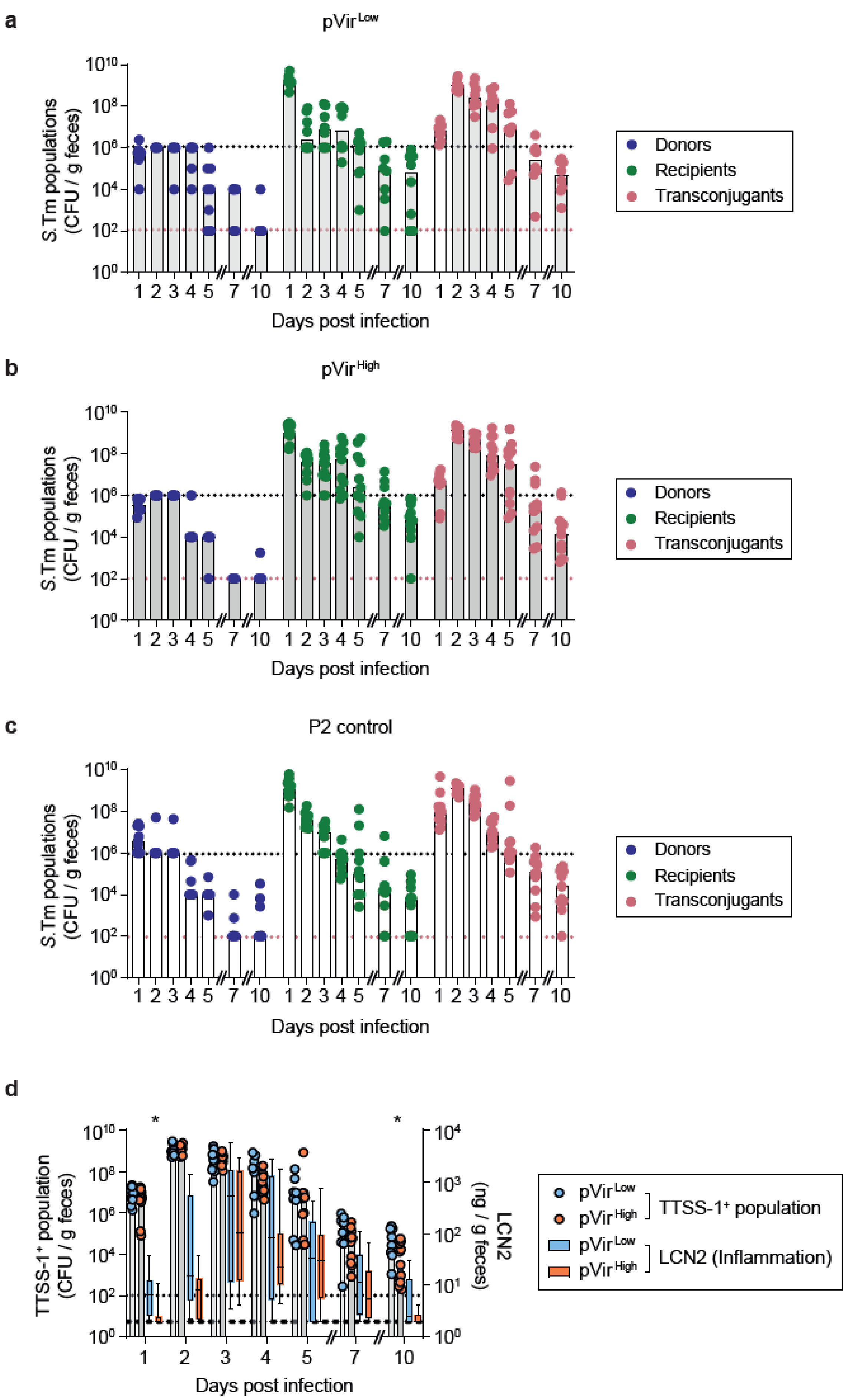
a
pVir Low
S.Tm populations
(CFU / g feces)
Days post infection
Donors
Recipients
Transconjugants
b
pVir High
S.Tm populations
(CFU / g feces)
Days post infection
Donors
Recipients
Transconjugants
c
P2 control
S.Tm populations
(CFU / g feces)
Days post infection
Donors
Recipients
Transconjugants
d
TTSS-1+ population
(CFU / g feces)
LCN2
(ng / g feces)
Days post infection
pVir Low
pVir High
TTSS-1+ population
pVir Low
pVir High
LCN2 (Inflammation)

Supplementary Figure S3. Fecal bacterial population sizes in samples and correlation with inflammation from experiments in figure 1. Fecal loads of donors (purple; Cm^R), recipients (green; Kan^R), and transconjugants (pale red; Cm^R, Kan^R) determined by selective plating. Replica plating was used to determine exact ratios of transconjugants compared to either donors or recipients. The black dotted line indicates the conservative detection limit for donors and recipients (depending on the dilution used for replica plating, values can appear below this line), and the pale red dotted line indicates the detection limit for transconjugants. Each data point is represented and bars indicate the median. Source data are provided as a Source Data file. **A)** Mice infected with *S.*Tm donors carrying $pVir^{Low}$ (n=8 mice). **B)** Mice infected with *S.*Tm donors carrying $pVir^{High}$ (n=11 mice). **C)** Mice infected with *S.*Tm donors with P2 (n=11 mice). **D)** The size of the population able to express TTSS-1 correlates with inflammatory state of the mouse (data from Figure 1; n=8 for $pVir^{Low}$ (blue) and n=11 for $pVir^{High}$ (orange). The transconjugant population size was multiplied by the proportion of cooperating clones (from **Fig. 1E**) to determine the size of the population able to express TTSS-1 (TTSS-1^+ population; plotted on the left y-axis; all data points plotted and line indicates the median). The black dotted line indicates the detection limit from selective plating. The inflammatory state is shown as a box (median indicated with a line and the quartiles are defined by the edges of the box) and whiskers define the minimum and maximum values (same data as in **Fig. 1C**). The dashed black line indicates the detection limit. $pVir^{High}$ was compared to $pVir^{Low}$ using a two-tailed Mann-Whitney U test ($p>0.05$ (ns), $p<0.05$ (*), $p<0.01$ (**), $p<0.001$ (***), $p<0.0001$ (****)).

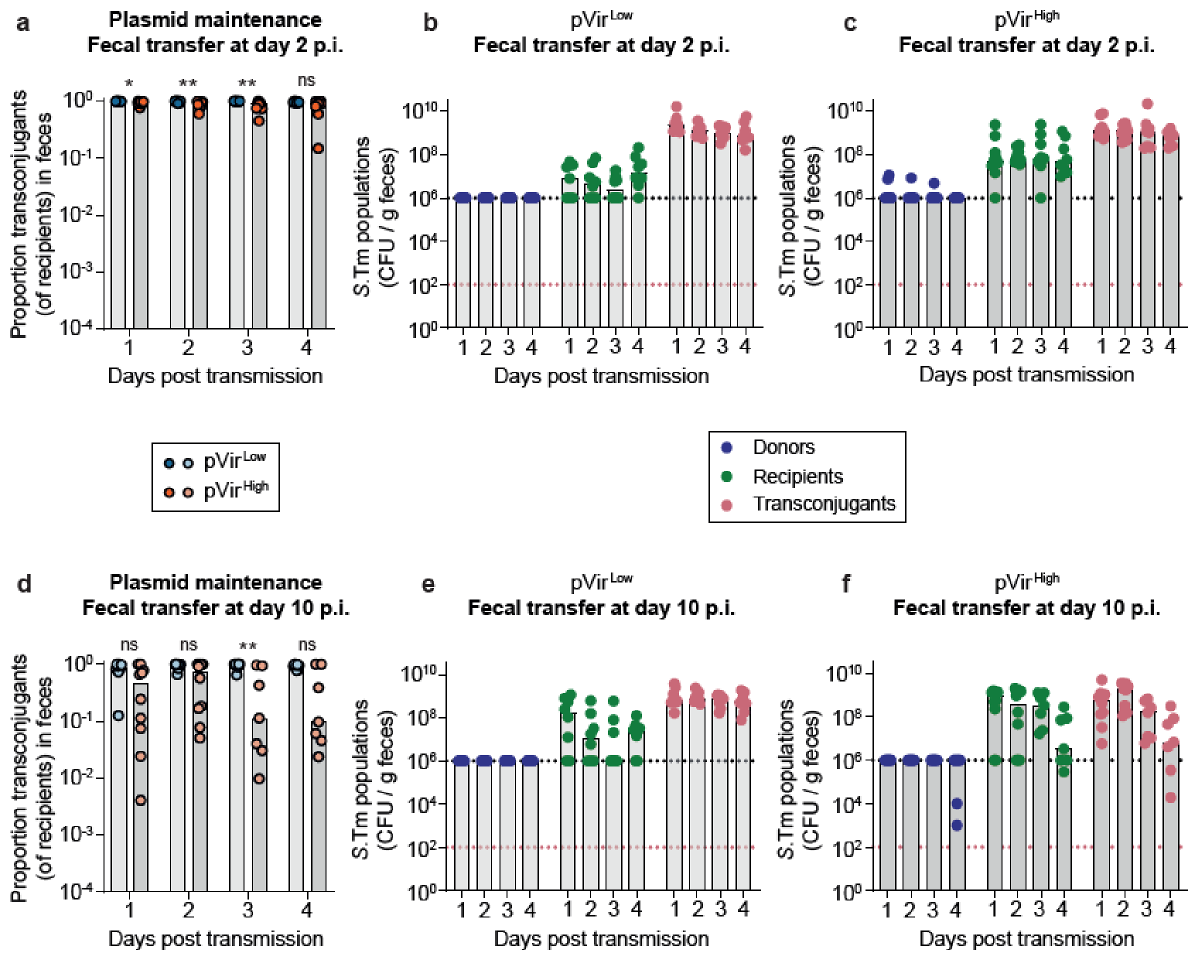

Supplementary Figure S4. Plasmid maintenance and fecal bacterial population sizes of mice in figure 2. A-C) Mice transmitted with fecal suspensions from day 2 post infection (n=8 for pVir^{Low} and n=11 for pVir^{High}). **D-F)** Mice transmitted with fecal suspensions from day 10 post infection (n=8 for pVir^{Low} and n=10 for pVir^{High}). **A,D)** Plasmid transfer was measured by selective plating: donors Cm^{R}, recipients Kan^{R}, and transconjugants both Cm^{R} and Kan^{R}. The proportion of transconjugants is calculated by dividing the transconjugant population by the sum of recipients and transconjugants. All data points are shown and medians are indicated by bars. Replica plating was used to determine exact ratio of transconjugants compared to recipients. Mice given fecal resuspensions with *S.*Tm harbouring pVir^{Low} (blue; dark shade for day 2 transmission; light shade for day 10 transmission) are compared to pVir^{High} (orange; dark shade for day 2 transmission; light shade for day 10 transmission) using a two-tailed Mann-Whitney U test ($p>0.05$ (ns), $p<0.05$ (*), $p<0.01$ (**), $p<0.001$ (***), $p<0.0001$ (****)). **B,C,E,F)** Fecal loads of donors (purple), recipients (green), and transconjugants (pale red) determined by selective plating. The black dotted line indicates the conservative detection limit for donors and recipients (depending on the dilution used for replica plating, values can appear below this line), and the pale red dotted line indicates the detection limit for transconjugants. Each data point is represented and bars indicate the median. **B,E)** Mice transmitted with fecal suspensions containing *S.*Tm with pVir^{Low}. **C,F)** Mice transmitted with fecal suspensions containing *S.*Tm with pVir^{High}. Source data for all panels are provided as a Source Data file.

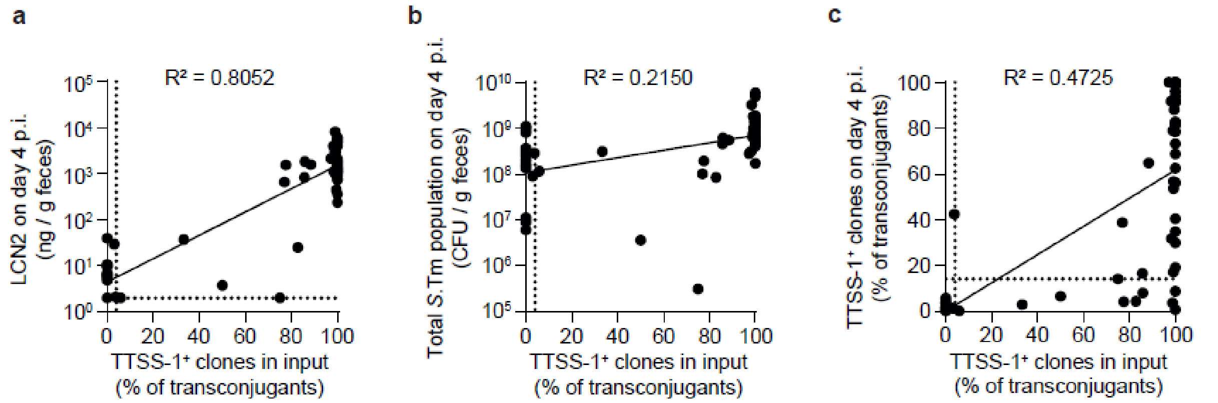

Supplementary Figure S5. Correlation between the proportion of cooperative clones given to mice and disease outcome. The proportion of cooperative clones (TTSS-1$^+$ clones) given to mice (x-axis; determined from **Fig. 1E**; assumed to be 100% for a TTSS-1$^+$ evolved clone and 0% for a TTSS-1$^-$ evolved clone) is plotted against the resulting inflammation (**panel A**), shedding population (**panel B**), and proportion of cooperative clones (**panel C**) at day 4 post infection in mice from **Fig. 2** and **Fig. 3** (y-axis). A linear regression was performed (y-axis log transformed in **panels A,B**) and was significantly non-zero as determined with an F test ($p<0.0001$ for **panels A,C**; $p=0.0002$ for **panel B**). The line of best fit and the goodness of fit (R^2) is shown on the graphs. Dotted lines indicated the detection limits. For detection limits based on colony blots, the conservative detection limit is shown, which is dependent on the number of colonies on the plate (values can therefore appear below the detection limit). Source data are provided as a Source Data file.

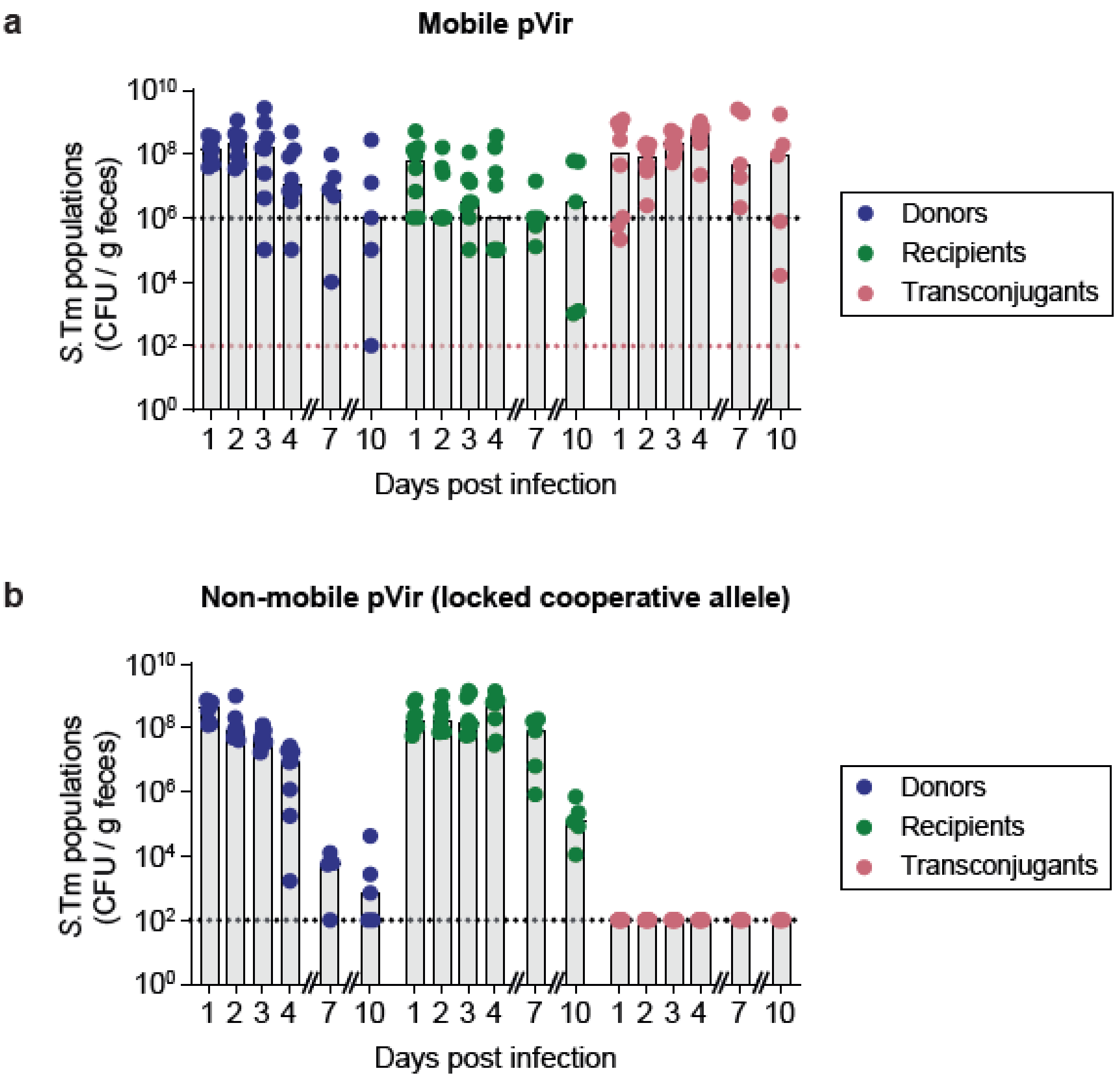

Supplementary Figure S6. Fecal bacterial population sizes in samples from experiments in figure 4. Fecal loads of donors (purple; Cm^R), recipients (green; Kan^R), and transconjugants (pale red; Cm^R, Kan^R) determined by selective plating. Replica plating was used to determine exact ratios of transconjugants compared to either donors or recipients. Each data point is represented and bars indicate the median. Source data are provided as a Source Data file. **A)** Mice infected with S.Tm donors and recipients in the mobile scenario (n=8 until day 4, n=5 until day 10). The black dotted line indicates the conservative detection limit for donors and recipients (depending on the dilution used for replica plating, values can appear below this line), and the pale red dotted line indicates the detection limit for transconjugants. **B)** Mice infected with S.Tm donors and recipients in the non-mobile scenario (n=8 until day 4, n=5 until day 10). The black dotted line indicates the detection limit.

	Clone	Ancestral donor pVir^{Low}	Z2296	Z2298	Z2299	Z2301	Z2302	Z2304	Z2305	Z2306	Z2308	Z2309	Z2310
	pVir type	Low	Low	Low	Low	Low	Low	Low	Low	Low	Low	Low	Low
	Day isolated	N/A	7	7	7	7	7	7	10	10	10	7	7
	SipC phenotype	-	+	-	+	-	+	-	-	+	+	-	+
	Mouse	N/A	AL876	AL876	AL877	AL877	AL878	AL878	AM128	AM128	AM129	AM130	AM130
pVir mutations	Annotated function												
hilD	transcriptional regulator HilD			285 bp Indel		134 bp Indel		409 bp Indel	100 bp Indel			365 bp Indel G>A (pos 584 in CDS)	

Table S1. Summary of SNPs or indels in pVir^{Low} in evolved transconjugants. For all clones, the SipC phenotype from colony blot (as a proxy for TTSS-1expression; + indicates positive and blue fill labels clones of that phenotype, - indicates negative and yellow fill labels clones of that phenotype) is shown and the mutations are summarized. If blank, the region of the genome contains no mutations. The *hilD* genotype is highlighted in either blue or yellow fill according to the SipC phenotype to allow for clear correlation. Blue: SipC positive clones; Yellow: SipC negative clones. Pos. position; CDS: Coding sequence. Indel: Insertion/deletion.

	Clone	Ancestral donor pVir^{High}	Z2238	Z2239	Z2242	Z2243	Z2244	Z2245	Z2246	Z2247	Z2252	Z2253	Z2254	Z2255	Z2311	Z2312
	pVir type	High	High	High	High	High	High	High	High	High	High	High	High	High	High	High
	Day isolated	N/A	10	10	10	10	10	10	10	10	10	10	10	10	10	10
	SipC phenotype	-	+	-	+	-	+	-	+	-	-	+	-	-	-	+
	Mouse	N/A	AG58	AG60	AG300	AG300	AG301	AG301	AG302	AG302	AG590	AG591	AG591	AG592	AM132	AM132
pVir mutations	Annotated function															
hilD	transcriptional regulator HilD			5 bp Indel		63 bp Indel		63 bp Indel		G > A (pos 173 in CDS)				133 bp Indel	258 bp Indel	
Upstream of *hilD*	transcriptional regulator HilD										310 bp Indel		326 bp Indel			
SL1344_RS24670	Transcription termination factor NusG				G > T (pos 415 in CDS)	G > T (pos 415 in CDS)	G > T (pos 415 in CDS)	G > T (pos 415 in CDS)	G > T (pos 415 in CDS)							

Table S2. Summary of SNPs or indels in pVir^{High} in evolved transconjugants. For all clones, the SipC phenotype from colony blot (as a proxy for TTSS-1expression; + indicates positive and blue fill labels clones of that phenotype, - indicates negative and yellow fill labels clones of that phenotype) is shown and the mutations are summarized. If blank, the region of the genome contains no mutations. The *hilD* genotype is highlighted in either blue or yellow fill according to the SipC phenotype to allow for clear correlation. Blue: SipC positive clones; Yellow: SipC negative clones. Pos. position; CDS: Coding sequence. Indel: Insertion/deletion.

	Clone	Ancestral donor pVir^{Low}	Ancestral recipient	Z2296	Z2298	Z2299	Z2301	Z2302	Z2304	Z2305	Z2306	Z2308	Z2309	Z2310
	**pVir type**	Low	N/A	Low	Low	Low	Low	Low	Low	Low	Low	Low	Low	Low
	**Day isolated**	N/A	N/A	7	7	7	7	7	7	10	10	7	7	7
	**SipC phenotype**	-	-	+	-	+	-	+	-	-	+	+	-	+
	**Mouse**	N/A	N/A	AL876	AL876	AL877	AL877	AL878	AL878	AM128	AM128	AM129	AM130	AM130
**Chromosomal mutations in CDS not present in the 14028 ancestor**	**Annotated function**													
melR	transcriptional regulator MelR				4554843 G > A									
melB	melbiose:sodium transporter MelB			4558548 G > -		4558548 G > -	4558548 G > -	4558548 G > -						
ssaV	SPI-2 type III secretion system apparatus protein SsaV	Δ	Δ	Δ	Δ	Δ	Δ	Δ	Δ	Δ	Δ	Δ	Δ	Δ
hilD	transcriptional regulator HilD	Δ	Δ	Δ	Δ	Δ	Δ	Δ	Δ	Δ	Δ	Δ	Δ	Δ
sipA	SPI-1 type III secretion system effector SipA		3046007 A > G	3046007 A > G	3046007 A > G	3046007 A > G	3046007 A > G	3046007 A > G	3046007 A > G	3046007 A > G	3046007 A > G	3046007 A > G	3046007 A > G	3046007 A > G
invG	SPI-1 type III secretion system outer membrane ring protein InvG	Δ												

Table S3. Summary of SNPs or indels in coding sequences of the chromosome of evolved transconjugants with pVir^{Low}. For all clones, the SipC phenotype from colony blot (as a proxy for TTSS-1 expression; + indicates positive and blue fill labels clones of that phenotype, - indicates negative and yellow fill labels clones of that phenotype) is shown and the mutations are summarized. For variants that affect single nucleotides, the position in the 14028S reference chromosome (NCBI accession NC_016856.1) is indicated along with the allele change. For targeted deletions introduced by allelic replacement or P22 transduction, the "Δ" symbol is used. Variants are only shown if they occurred in >70% of reads. Mutations are excluded if they also occurred in our ancestral lab strain of 14028S. If blank, the region of the genome contains no mutations. Blue: SipC positive clones; Yellow: SipC negative clones; Δ: Locus removed by in-frame deletion; N/A: Non Applicable; Pos. position; CDS: Coding sequence. Indel: Insertion/deletion.

	Clone	Ancestral donor pVir^{High}	Ancestral recipient	Z2238	Z2239	Z2242	Z2243	Z2244	Z2245	Z2246	Z2247	Z2252	Z2253	Z2254	Z2255	Z2311	Z2312
	pVir type	High	N/A	High	High	High	High	High	High	High	High	High	High	High	High	High	High
	Day isolated	N/A	N/A	10	10	10	10	10	10	10	10	10	10	10	10	10	10
	SipC phenotype	-	-	+	-	+	-	+	-	+	-	-	+	-	-	-	+
	Mouse	N/A	N/A	AG58	AG60	AG300	AG300	AG301	AG301	AG302	AG302	AG590	AG591	AG591	AG592	AM132	AM132
**Chromosomal mutations in CDS not present in the 14028 ancestor**	**Annotated function**																
melR	transcriptional regulator MelR											4555269 T > G					
melB	melbiose:sodium transporter MelB			4558394 C > T			4558406 T > G		4558406 T > G				4558403 G > A		4558406 T > G		4558156 T > A
ssaV	SPI-2 type III secretion system apparatus protein SsaV	Δ	Δ	Δ	Δ	Δ	Δ	Δ	Δ	Δ	Δ	Δ	Δ	Δ	Δ	Δ	Δ
hilD	transcriptional regulator HilD	Δ	Δ	Δ	Δ	Δ	Δ	Δ	Δ	Δ	Δ	Δ	Δ	Δ	Δ	Δ	Δ
sipA	SPI-1 type III secretion system effector SipA		3046007 A > G	3046007 A > G	3046007 A > G	3046007 A > G	3046007 A > G	3046007 A > G	3046007 A > G	3046007 A > G	3046007 A > G	3046007 A > G	3046007 A > G	3046007 A > G	3046007 A > G	3046007 A > G	3046007 A > G
invG	SPI-1 type III secretion system outer membrane ring protein InvG	Δ															
tatD	3'-5' ssDNA/RNA exonuclease TatD																4196195 T > G

Table S4. Summary of SNPs or indels in coding sequences of the chromosome of evolved transconjugants with pVir^{High}. For all clones, the SipC phenotype from colony blot (as a proxy for TTSS-1 expression; + indicates positive and blue fill labels clones of that phenotype, - indicates negative and yellow fill labels clones of that phenotype) is shown and the mutations are summarized. For variants that affect single nucleotides, the position in the 14028S reference chromosome (NCBI accession NC_016856.1) is indicated along with the allele change. For targeted deletions introduced by allelic replacement or P22 transduction, the "Δ" symbol is used. Variants are only shown if they occurred in >70% of reads. Mutations are excluded if they also occurred in our ancestral lab strain of 14028S. If blank, the region of the genome contains no mutations. Blue: SipC positive clones; Yellow: SipC negative clones; Δ: Locus removed by in-frame deletion; N/A: Non Applicable; Pos. position; CDS: Coding sequence. Indel: Insertion/deletion.
