# Supplementary material for: Impact of horizontal gene transfer on emergence and stability of cooperative virulence in Salmonella Typhimurium
Source: Nat Commun. 2022 Apr 11;13:1939. doi: 10.1038/s41467-022-29597-7 (PMC9001671; doi:10.1038/s41467-022-29597-7)
Supplement: Supplementary file 3 — Reporting Summary [file 41467_2022_29597_MOESM3_ESM.pdf]

nature research

Corresponding author(s): Médéric Diard and Wolf-Dietrich Hardt

Last updated by author(s): Mar 3, 2022

Reporting Summary

Nature Research wishes to improve the reproducibility of the work that we publish. This form provides structure for consistency and transparency in reporting. For further information on Nature Research policies, see our Editorial Policies and the Editorial Policy Checklist.

Statistics

For all statistical analyses, confirm that the following items are present in the figure legend, table legend, main text, or Methods section.

n/a	Confirmed	
☐	☒	The exact sample size (*n*) for each experimental group/condition, given as a discrete number and unit of measurement
☐	☒	A statement on whether measurements were taken from distinct samples or whether the same sample was measured repeatedly
☐	☒	The statistical test(s) used AND whether they are one- or two-sided<br>*Only common tests should be described solely by name; describe more complex techniques in the Methods section.*
☐	☒	A description of all covariates tested
☐	☒	A description of any assumptions or corrections, such as tests of normality and adjustment for multiple comparisons
☐	☒	A full description of the statistical parameters including central tendency (e.g. means) or other basic estimates (e.g. regression coefficient) AND variation (e.g. standard deviation) or associated estimates of uncertainty (e.g. confidence intervals)
☒	☐	For null hypothesis testing, the test statistic (e.g. *F*, *t*, *r*) with confidence intervals, effect sizes, degrees of freedom and *P* value noted<br>*Give P values as exact values whenever suitable.*
☒	☐	For Bayesian analysis, information on the choice of priors and Markov chain Monte Carlo settings
☒	☐	For hierarchical and complex designs, identification of the appropriate level for tests and full reporting of outcomes
☒	☐	Estimates of effect sizes (e.g. Cohen's *d*, Pearson's *r*), indicating how they were calculated

Our web collection on statistics for biologists contains articles on many of the points above.

Software and code

Policy information about availability of computer code

Data collection	Data were collected using Microsoft Excel 2016 (16.0.5266.1000)
Data analysis	Statistical tests on experimental data were performed using GraphPad Prism 8.0.2 for Windows. Sequences were analyzed using CLC Genomic Workbench 12.0.2.

For manuscripts utilizing custom algorithms or software that are central to the research but not yet described in published literature, software must be made available to editors and reviewers. We strongly encourage code deposition in a community repository (e.g. GitHub). See the Nature Research guidelines for submitting code & software for further information.

Data

Policy information about availability of data

All manuscripts must include a data availability statement. This statement should provide the following information, where applicable:

- Accession codes, unique identifiers, or web links for publicly available datasets
- A list of figures that have associated raw data
- A description of any restrictions on data availability

All raw data are available on request to the corresponding authors without restriction. Citation of the current paper should accompany any further publication based on these data. Figures 1 to 4 and S1 to S6 are associated with data provided in the source data Excel sheet.
The S.Tm 14028S reference chromosome is accessible under the NCBI accession number NC_016856.1.
The S.Tm SL1344 reference chromosome is accessible under the NCBI accession number FQ312003.1.

Field-specific reporting

Please select the one below that is the best fit for your research. If you are not sure, read the appropriate sections before making your selection.

- [x] Life sciences
- [ ] Behavioural & social sciences
- [ ] Ecological, evolutionary & environmental sciences

For a reference copy of the document with all sections, see nature.com/documents/nr-reporting-summary-flat.pdf

Life sciences study design

All studies must disclose on these points even when the disclosure is negative.

Sample size	Sample size for mouse experiments was typically estimated based on a One-way ANOVA analysis with an effect size of between 0.5 and 2 and a power of 90% (note that final analysis was typically non-parametric or required two-way ANOVA, but this system was used to give simple conservative estimates). Where large effects were expected, we followed the convention of using at least 5 mice per group, pooled from at least two separate experiments.<br>No sample size was calculated for in vitro measurements of expression and cost of ttss-1 in strains harboring pVir plasmids (figure S1). Expression and growth dynamics presented in figure S1 were reproducible enough between independent clones and repetitions to detect significant variations between conditions. Moreover, controls involving reference S.Tm strains (WT and hilD mutant), well characterized in previous publications, further validated these results.
Data exclusions	No data were excluded in this study
Replication	All data shown is the result of pooling across at least two separate experiments with multiple cages of mice represented in each group.
Randomization	Animals were randomized to experimental groups by cage, ensuring approximately equal gender distribution between the groups by pooling across multiple experiments.<br>For in vitro experiments (figure S1), several clones of strains harboring the different pVir plasmids were randomly picked after construction in order to perform independent biological replicates.
Blinding	Experimenters were not blinded because the acquisition of the datasets could not be influenced by unconscious biases during experiments or subjective readouts.

Reporting for specific materials, systems and methods

We require information from authors about some types of materials, experimental systems and methods used in many studies. Here, indicate whether each material, system or method listed is relevant to your study. If you are not sure if a list item applies to your research, read the appropriate section before selecting a response.

Materials & experimental systems

n/a	Involved in the study	
[ ]	[x]	Antibodies
[x]	[ ]	Eukaryotic cell lines
[x]	[ ]	Palaeontology and archaeology
[ ]	[x]	Animals and other organisms
[x]	[ ]	Human research participants
[x]	[ ]	Clinical data
[x]	[ ]	Dual use research of concern

Methods

n/a	Involved in the study	
[x]	[ ]	ChIP-seq
[x]	[ ]	Flow cytometry
[x]	[ ]	MRI-based neuroimaging

Antibodies

Antibodies used	Rabbit anti-SipC polyclonal serum provided by Virotech Diagnostics GmbH (reference number: VT110712)
Validation	The specificity of this antibody for the protein SipC produced by S.Tm has been validated in several reports including:<br><br>Diard, M. et al. Stabilization of cooperative virulence by the expression of an avirulent phenotype. Nature 494, 353-356, doi:10.1038/nature11913 (2013).<br>and<br>Bakkeren, E., Dolowschiak, T. & Diard, M. Detection of Mutations Affecting Heterogeneously Expressed Phenotypes by Colony Immunoblot and Dedicated Semi-Automated Image Analysis Pipeline. Front Microbiol 8, 2044, doi:10.3389/fmicb.2017.02044 (2017).<br><br>In the current manuscript, sequencing of clones sorted according to SipC detection by Colony Blot using this serum validated that mutations in hilD were responsible for the SipC negative phenotype (supplementary tables S1 to S4) as previously published.

Animals and other organisms

Policy information about studies involving animals; ARRIVE guidelines recommended for reporting animal research

Laboratory animals	8-12 week old specified opportunistic pathogen free (SOPF) C57BL/6J mice male or female.
Wild animals	No wild animals were used in the study
Field-collected samples	No field collected samples were used in the study
Ethics oversight	All infection experiments were approved by the responsible authorities (Tierversuchskommission, Kantonales Veterinäramt Zürich, licenses 193/2016 and 158/2019).

Note that full information on the approval of the study protocol must also be provided in the manuscript.
